# Supplementary material for: Validation of an Italian Questionnaire of Adherence to the Ketogenic Dietary Therapies: iKetoCheck
Source: Foods. 2023 Aug 26;12(17):3214. doi: 10.3390/foods12173214 (PMC10486753; doi:10.3390/foods12173214)
Supplement: Supplementary file 1 [file foods-12-03214-s001.zip › foods-2474598-supplementary.pdf]

foods

MDPI

Supplementary

Table S1: Covariance matrix (polychromic correlation) of iKetoCheck.

**Question**	1	2	3	4	5	6	7	8	9	10
**q1**	1.000									
**q2**	0.658	1.000								
**q3**	0.364	0.235	1.000							
**q4**	0.462	0.508	0.263	1.000						
**q5**	0.313	0.335	0.009	0.557	1.000					
**q6**	0.190	-0.012	0.256	0.098	0.129	1.000				
**q7**	0.608	0.479	0.182	0.249	0.514	0.096	1.000			
**q8**	0.196	0.232	0.664	0.358	0.219	0.359	0.128	1.000		
**q9**	0.316	0.238	0.282	0.296	0.461	0.111	0.515	0.362	1.000	
**q10**	0.180	0.172	0.321	0.073	0.227	0.227	0.255	0.473	0.216	1.000

Supplementary

Table S2: iKetoCheck: Assessment tool of adherence to the ketogenic diet therapies

**iKetoCheck - KETOGENIC THERAPIES ADHERENCE'S TOOL**<br>Below you will find 10 sentences. We ask you to indicate one of the options from 1 to 5, according to how often the above statements occur. These statements concern your experience regarding the management of Ketogenic Diet Therapy followed by your family member or by yourself.	
1. I shared with family members/acquaintances/school/work the characteristics of the diet and the need not to administer/take foods other than prescribed	1. I never do<br>2. I do it little<br>3. I do sometimes<br>4. I do this often<br>5. I always do it
2. I organized myself to prepare meals that allow you to follow the diet even outside the home	1. I never do<br>2. I do it little<br>3. I do sometimes<br>4. I do this often<br>5. I always do it
3. I measure the levels of ketosis according to the indications of my reference center	1. I never do<br>2. I do it little<br>3. I do sometimes<br>4. I do this often<br>5. I always do it
4. Weigh all foods according to the prescribed diet	1. I never do<br>2. I do it little<br>3. I do sometimes<br>4. I do this often<br>5. I always do it
5. I only eat foods allowed in the dietary prescription	1. I never do<br>2. I do it little<br>3. I do sometimes<br>4. I do this often<br>5. I always do it
6. All medicines and supplements used are replaced, where possible, with similar sugar-free products	1. I never do<br>2. I do it little<br>3. I do sometimes<br>4. I do this often<br>5. I always do it
7. I show up for all scheduled appointments with the nutrition team and neurologist/neuropsychiatrist	1. I never do<br>2. I do it little<br>3. I do sometimes<br>4. I do this often<br>5. I always do it
8. I fill out the monitoring form (for example Ketonemia diary) according to the instructions of the caregiver (doctor/dietitian/nutritionist)	1. I never do<br>2. I do it little<br>3. I do sometimes<br>4. I do this often<br>5. I always do it
9. I read the nutritional labels (carbohydrates, proteins and fats) on all packaged products and ask, when in doubt, to the nutritionist / dietitian if their use is permitted before consuming it	1. I never do<br>2. I do it little<br>3. I do sometimes<br>4. I do this often<br>5. I always do it
10. I cook at home and talk to the specialist to validate the new recipes that I find	1. I never do<br>2. I do it little<br>3. I do sometimes<br>4. I do this often<br>5. I always do it

Supplementary

Table S3. Characteristics of the patients who answered the iKetocheck.

**Characteristics**	**n (%)**
**Age (yrs)**	
0-2	9 (7,9)
3-12	66 (57,9)
13-20	29 (25,4)
>20	10 (8,8)
**Total**	114 (100,0)
**Diagnosis**	
Glut 1 Syndrome	64 (56,1)
Drug-resistant epilepsy	35 (30,7)
Migraine	1 (0,9)
Others	14 (12,3)
**Total**	114 (100,0)
**Diet duration (months)**	
<6	8 (7,3)
6-12	16 (14,5)
12-24	22 (20,0)
<24	64 (58,2)
**Total**	110 (100,0)
**Type of KDT**	
cKDT	93 (81,6)
MCT	16 (14,0)
MAD	4 (3,5)
LGIT	1 (0,9)
**Total**	114 (100,0)
**Mean ketonemia referred by patients (mmol/dL)**	
<2	29 (26,4)
2-5	80 (72,7)
>5	1 (0,9)
**Total**	110 (100,0)
**Perceived efficacy on symptoms (%)**	
0-25	4 (3,5)
25-50	13 (11,4)
50-75	46 (40,4)
75-100	51 (44,7)
**Total**	114 (100,0)

Supplementary

Table S4. Test of reproducibility according to Pearson correlation (n= 61).

Question	1_a	2_a	3_a	4_a	5_a	6_a	7_a	8_a	9_a	10_a	Score_1
1_b	0,660*										
2_b		0,655*									
3_b			0,764*								
4_b				0,708*							
5_b					0,535*						
6_b						0,486*					
7_b							0,710*				
8_b								0,738*			
9_b									0,337*		
10_b										0,736*	
Score_2											0,849*

* Pearson correlation test.

Table S5. Test of reproducibility according to Pearson correlation of the whole sample (n=73).

Question	1_a	2_a	3_a	4_a	5_a	6_a	7_a	8_a	9_a	10_a	Score_1
1_b	,442**										
2_b		,655**									
3_b			,677**								
4_b				,456**							
5_b					,390**						
6_b						,439**					
7_b							,472**				
8_b								,694**			
9_b									,348**		
10_b										,599**	
Score_2											,587**
